# Echo Intensity Correction Method for Ultrasound Computed Tomography in Musculoskeletal Imaging

**DOI:** 10.3390/bioengineering13030352

**Published:** 2026-03-18

**Authors:** Junchao Zeng, Ding Lou, Qin Zhang, Hui Zhang, Hongyi Zhu, Xing Cheng, Tengfei Wang, Sanping Xu, Yan Ling, Mingyue Ding

**Affiliations:** 1 Health Management Center, Union Hospital, Tongji Medical College, Huazhong University of Science and Technology, Wuhan 430022, China; d202380995@hust.edu.cn (J.Z.); zhy2830533404@163.com (H.Z.);; 2 Medical Ultrasound Laboratory, Department of Biomedical Engineering, College of Life Science and Technology, Advanced Bio-Medical Imaging Facility, Huazhong University of Science and Technology, Wuhan 430074, China; d202380973@hust.edu.cn; 3 Medical Equipment Department, Union Hospital, Tongji Medical College, Huazhong University of Science and Technology, Wuhan 430022, China; louding153102@163.com; 4 Rehabilitation Medicine Department, Jingmen Central Hospital, Jingmen 448001, China; zqtcm@126.com; 5 Department of Biomedical Engineering, Chongqing Hospital Union Hospital, Tongji Medical College, Huazhong University of Science and Technology, Chongqing 401100, China; ti_3601@163.com

**Keywords:** ultrasound computed tomography, musculoskeletal diseases, echo intensity correction

## Abstract

Ultrasound computed tomography (USCT) has emerged as a promising tool for quantitative assessment of musculoskeletal (MSK) diseases. However, the accuracy of echo intensity—a key imaging biomarker—is often compromised by non-optimal imaging conditions, such as probe tilt and limb eccentricity. In this study, we propose a novel echo intensity correction method for USCT that quantitatively compensates for these two major sources of error. The method integrates finite element simulation and phantom experiments to establish correction functions for each influencing factor. These functions are then applied to USCT images from volunteers through automated parameter extraction and intensity adjustment. Validation on both phantom and in vivo data showed that the proposed method significantly improved the uniformity and diagnostic accuracy of echo intensity measurements, leading to a clear improvement in the diagnostic accuracy of MSK diseases. This method enhances the reliability of USCT-based quantitative diagnosis and holds strong potential for broader clinical adoption.

## 1. Introduction

Musculoskeletal disorders pose significant socioeconomic challenges worldwide, with an estimated 1.71 billion people affected globally [1,2]. Sarcopenia, a prevalent condition in this system, is a syndrome characterized by age-related reduction in skeletal muscle mass and function [3], associated with poor prognosis [4]. A recent systematic review indicates that the prevalence of sarcopenia ranges from 8–36% among young adults and 10–27% among the elderly, depending on diagnostic criteria [5].

Currently, traditional ultrasound (US) is the most commonly used diagnostic tool for musculoskeletal system disorders [6] and has been employed as a low-cost, minimally invasive option for diagnosing musculoskeletal diseases [7]. Studies indicate that ultrasound imaging can not only facilitate the early diagnosis and quantification of sarcopenia but also provide a comprehensive assessment of muscle health by evaluating parameters such as muscle structure, echo intensity, and elasticity [8]. Among these, echo intensity (EI) serves as a critical metric for assessing muscle mass in ultrasound [9], enabling effective evaluation of muscle health status. However, its application is significantly limited, as imaging results are highly dependent on operator experience, including precise control of probe angles and appropriate selection of scanning positions [10]. This leads to substantial variability in measurement outcomes between different operators or within the same operator at different times, resulting in poor reproducibility and hindering the implementation of standardized diagnostic protocols. The pursuit of standardized, automated quantitative imaging pipelines has become a research priority in clinical translational medical imaging, with deep learning and artificial intelligence emerging as core technologies to address the limitations of manual operation [11,12]. Therefore, there is an urgent need for novel diagnostic methods.

Ultrasound computed tomography (USCT) reconstructs images based on the transmission and reflection properties of ultrasound waves by tissues, providing three-dimensional information on mechanical properties such as sound velocity and density. Compared to conventional ultrasound, USCT employs full waveform inversion (FWI) technology to overcome diffraction limitations, significantly improving resolution [13]. It can clearly display the features of muscles, connective tissues, and bones, demonstrating high adaptability in detecting muscle atrophy (e.g., sarcopenia) or tendon pathologies [14].

**Figure 1:**
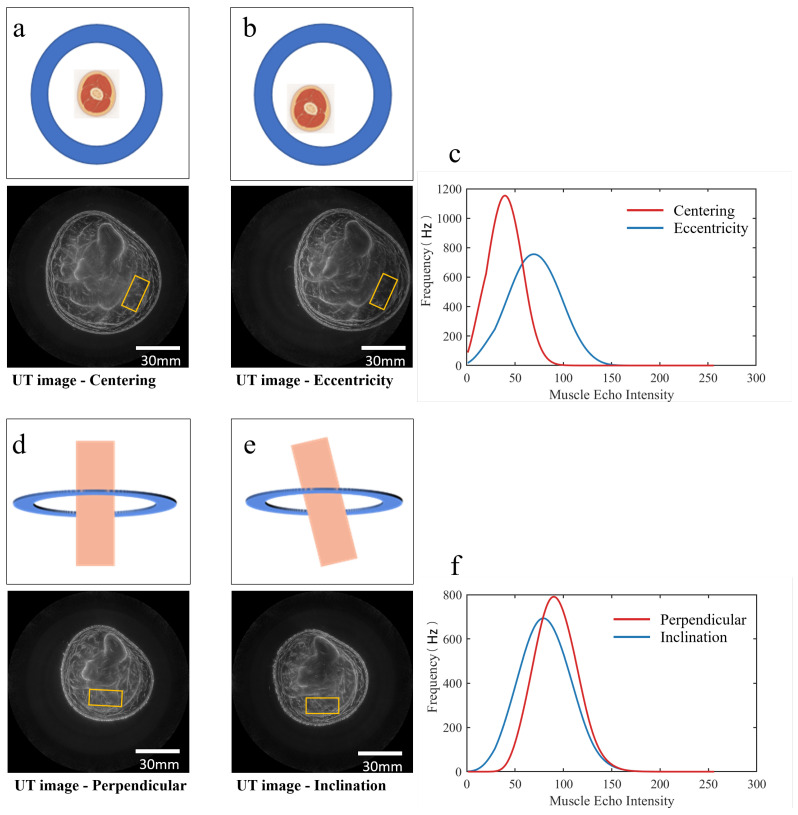
The Impact of deviation in Limb placement on muscle echo Intensity. The distribution of echo intensities (**c**) when the calf is positioned centrally (**a**) and eccentrically (**b**) in the scanning area. The distribution of echo intensities (**f**) when the calf is positioned in the perpendicular (**d**) and inclined orientations (**e**).

Therefore, to overcome the standardization bottleneck of traditional ultrasound, we applied echo intensity correction methods in USCT to achieve three-dimensional imaging of tissues through automated standardized scanning with a ring probe, thereby enabling precise diagnostic standards for skeletal muscle system disorders such as sarcopenia. When USCT is employed to investigate sarcopenia, deviation in limb placement during scanning may occur, which can adversely affect the accuracy of echo intensity (see Figure 1).

The ultrasound computed tomography (USCT) system utilized in this study comprises three core functional components: a 2048-element annular transducer probe(Huazhong University of Science and Technology, Wuhan, China), an ultrasonic transceiver system(Huazhong University of Science and Technology, Wuhan, China), and a high-performance computer (Lenovo Group Ltd., Beijing, China) [15]. The annular transducer probe, as the most critical component of the USCT system, is configured with 2048 elements, a center frequency of 3.2 MHz, a relative bandwidth of 70%, and an inner diameter of 222 mm. During scanning, the subject places their limbs inside the annular probe, and the waterproof transducer is immersed in a tank filled with warm water, which acts as the coupling medium. The ultrasonic transceiver system is responsible for high-voltage excitation of the annular probe and acquisition of echo signals, while the high-performance computer undertakes the reconstruction computation of the collected echo signals.

In this study, a synthetic aperture approach was employed for reflection USCT imaging, where each element of an annular transducer sequentially transmitted spherical waves while all elements simultaneously received ultrasonic echoes; low-resolution images for each transmit-receive element pair were reconstructed via the delay-and-sum (DAS) method, and these images were fused to achieve dynamic transmit and receive focusing, with the focal signal at each imaging point reconstructed using a weighted summation formula [16].

## 2. Materials and Methods

### 2.1. Identification of Influencing Factors

**Figure 2:**
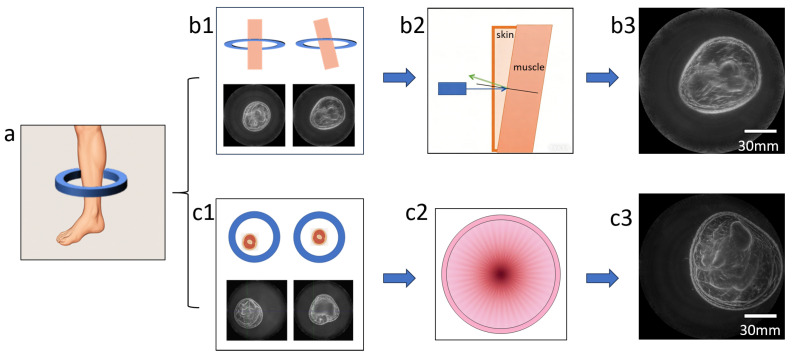
A schematic illustration of the process by which probe tilt angle and limb eccentricity affect echo intensity.Schematic diagram of USCT scanning of the calf (**a**); scanning processes and imaging results of the limb in normal and inclined states (**b1**); schematic diagram showing that part of the reflected waves cannot be received by the ultrasonic transducer due to the inclined state (**b2**); imaging effect in the inclined state with unclear edge imaging (**b3**); scanning processes and imaging effects of the limb in normal and eccentric states (**c1**); distribution of ultrasonic waves in the scanning area, showing a concentration toward the center (**c2**); imaging effect in the eccentric state, presenting “brighter in the center and darker at the edges” (**c3**).

Two primary factors were identified as major contributors to echo intensity variation in USCT imaging: Probe tilt angle: Deviation from the perpendicular incidence of the ultrasound beam. Limb eccentricity: Misalignment between the limb center and the scanner’s central axis. Each factor was varied systematically in both simulations and experiments to quantify their effects on echo intensity. Figure 2 shows the process by which these two factors affect echo intensity. During the automated scanning of the ring probe, when the calf tilts, part of the echo is reflected away, resulting in a weaker received echo intensity and thereby compromising USCT imaging quality. When misalignment occurs between the limb center and the scanner’s central axis, the image distribution is adversely affected.

### 2.2. Numerical Simulations

**Figure 3:**
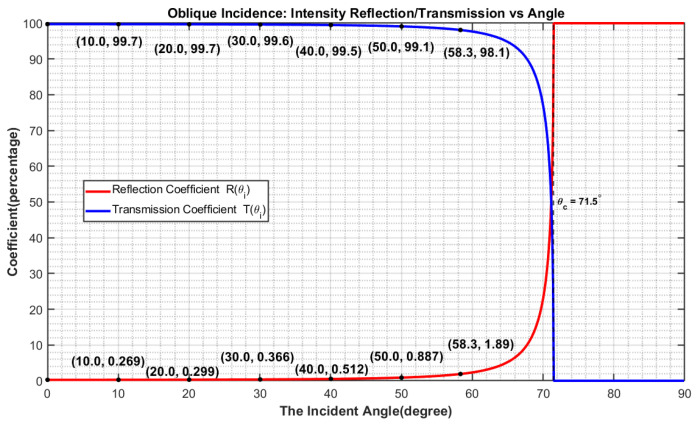
Relationship between Acoustic Wave Reflection/Transmission Intensities and Incident Angle under Inclined Incidence.

In ultrasonic computed tomography (USCT), the inclination of the measured object interface can lead to non-perpendicular incidence of acoustic waves, thereby affecting imaging quality. This experiment was carried out on the MATLAB 2023b platform (The MathWorks, Inc., Natick, MA, USA) to quantitatively analyze the influence of the inclined incident angle on the reflection and transmission intensities of acoustic waves, aiming to reveal the mechanism by which interface inclination affects acoustic energy distribution and imaging performance. The simulation results (see Figure 3) show that at small inclination angles, the acoustic transmission intensity remains at a high level with gentle changes. As the inclination angle approaches the critical angle, the transmission intensity drops sharply and the reflection intensity increases significantly, indicating that large inclination angles can notably reduce the effective penetration of acoustic waves.

**Figure 4:**
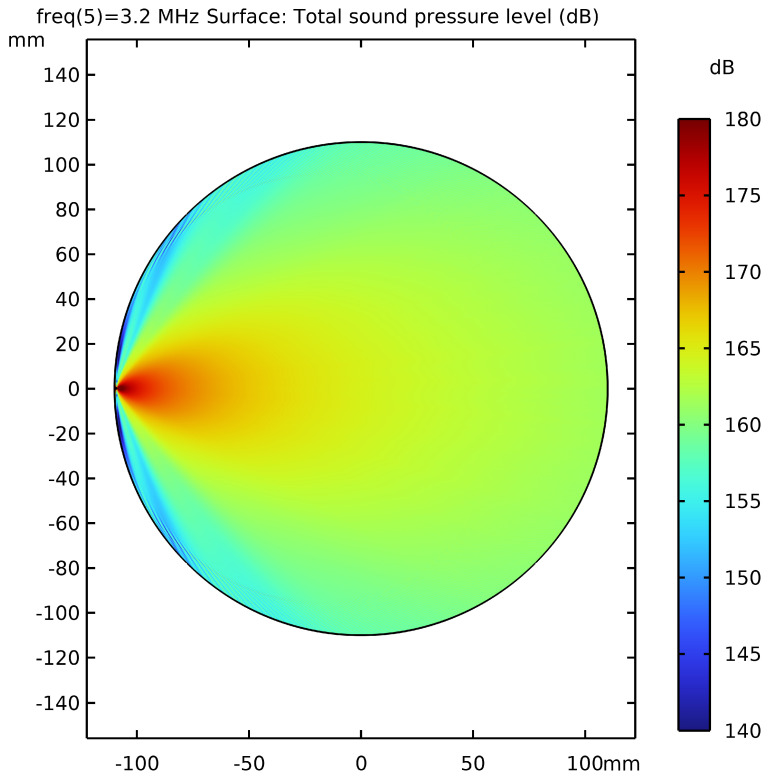
Directivity We have uploaded the revised high-resolution Figure 9. distribution of the ultrasonic transducer’s acoustic field at 3.2 MHz (dB).

This experiment conducted a simulation study on the acoustic field distribution characteristics of the transducer in an ultrasonic computed tomography (USCT) system, and numerically simulated the transducer’s acoustic field at an operating frequency of 3.2 MHz to reveal the mechanism by which the transducer’s directivity affects imaging artifacts. The simulation results (see Figure 4) clearly demonstrate the directivity characteristics of the transducer’s acoustic field: the acoustic energy is significantly concentrated in the area directly in front of the transducer and gradually attenuates toward the periphery. This non-uniform acoustic field superposition effect can introduce eccentric artifacts in the imaging results, leading to systematic bias in local echo intensity.

### 2.3. Theoretical Analysis and Compensation Method

#### 2.3.1. Analysis and Compensation Method of Eccentricity

**Figure 5:**
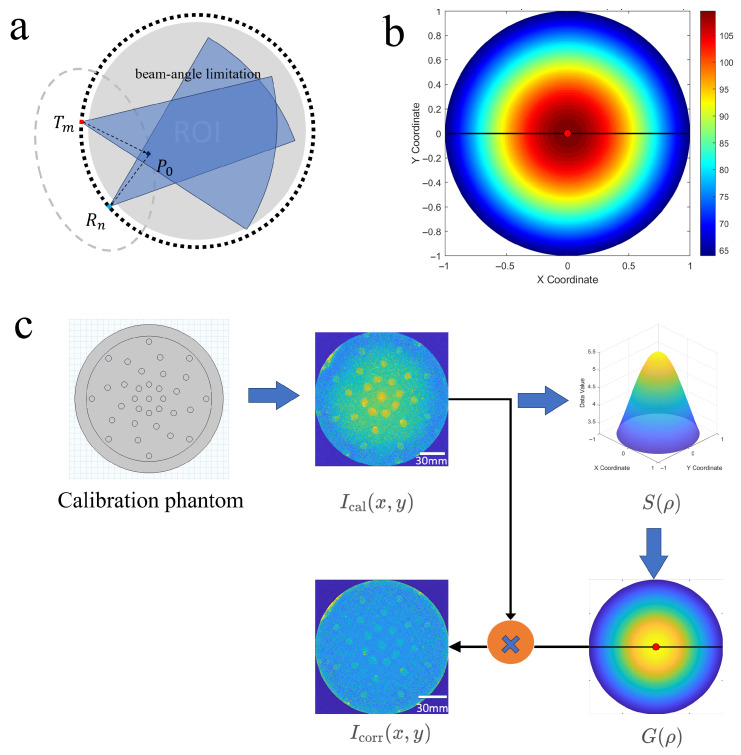
The principle, effect, and implementation workflow of radial sensitivity calibration and compensation in reflection-mode annular array imaging, where (**a**) shows the physical model of the annular array imaging system, demonstrating the beam-angle limitation and the effective observation path difference at different radial positions that cause radial sensitivity non-uniformity; (**b**) presents the original uncompensated imaging amplitude map, exhibiting the typical “brighter center, darker edge” radial intensity variation; and (**c**) depicts the complete “calibration–estimation–compensation” workflow, starting from the calibration phantom, extracting the radial sensitivity profile via annular averaging, constructing the radial gain compensation function, and applying it to obtain the corrected uniform image.

To correct the radial non-uniformity of “center brightening and edge darkening” in reflection-mode annular array imaging, Figure 5 shows the analysis and compensation method of eccentricity effect.we model this effect as the system’s radial sensitivity function S(ρ) (where ρ denotes the distance from a pixel to the center of the imaging circle) and employ a “calibration–estimation–compensation” workflow for correction. The underlying physical principle is that reflection-mode reconstruction essentially performs coherent accumulation of echo energy satisfying the elliptical equal-delay condition. Under the constraints of limited beam angle and transducer directivity, the effective observation paths at different radial positions exhibit systematic discrepancies from the single-channel echo amplitude, which results in an amplitude that varies with ρ. For nonlinear beamforming techniques such as DMAS, if inter-channel coherence is maintained at pixel *r* and the amplitude of each channel is approximately A(ρ), with the number of effective channels being $N_{\mathrm{eff}}\left(\rho \right)$, the output magnitude can be approximated as:

(1) IDMAS(ρ)∝Neff(ρ)2A2(ρ)

Thus, the radial sensitivity differences are amplified in the amplitude image, making them suitable for flexible compensation using a radial gain function.

Calibration Data Acquisition and Reconstruction: A frame of data from a uniform scatterer/stable reference reflector is acquired, following the identical transmission, reception, reconstruction, and post-processing pipeline (including DMAS parameters, filtering, and windowing functions) as in actual imaging. This yields the calibrated reconstruction $I_{\mathrm{cal}}\left(x,y\right)$. Compensation is performed in the linear amplitude domain (e.g., envelope amplitude or uncompressed amplitude map), which requires converting the imaging result to a linear domain first.

Radial Sensitivity Profile Estimation (Annular Averaging): Using the image center as the reference, we compute the radial average profile (annular averaging) to mitigate the impact of local structures.

Define the radial distance from a pixel to the center as:

(2) $$\rho \left(x,y\right)=\sqrt{x^{2}+y^{2}}$$

and the annular region at radius ρ as:

(3) $$\Omega \left(\rho \right)=\left\{(x,y):\rho -\Delta \leq \rho (x,y)<\rho +\Delta \right\}$$

The radial profile is then defined as:

(4) $$S\left(\rho \right)=\frac{1}{\left|\Omega (\rho )\right|}\sum_{\left(x,y)\in \Omega (\rho \right)}I_{\mathrm{cal}}\left(x,y\right)$$

We smooth/fit S(ρ) to obtain a continuous function $\tilde{S}\left(\rho \right)$ using a Savitzky–Golay filter [17], which performs local polynomial regression within a moving window to preserve peak characteristics while reducing high-frequency fluctuations. In this study, a third-order polynomial with a window length of 32 samples was applied to obtain a continuous function $\tilde{S}\left(\rho \right)$, and normalize it as:

(5) $$\hat{S}\left(\rho \right)=\frac{\tilde{S}\left(\rho \right)}{\tilde{S}\left(0\right)}$$

Construction and Application of Radial Gain Compensation Function: We define the radial gain function (with a numerical stabilization term ε to prevent excessive noise amplification):

(6) $$G\left(\rho \right)=\frac{1}{\hat{S}\left(\rho \right)}+\varepsilon$$

For any image I(x,y) to be corrected, we apply flexible radial compensation:

(7) $$I_{\mathrm{corr}}\left(x,y\right)=G\left(\rho (x,y)\right)\cdot I\left(x,y\right)$$

To avoid the amplification of edge noise and artifacts, we employ a clipping and masking strategy:

(8) $$G_{\mathrm{clip}}\left(\rho \right)=\left\{\begin{array}{cc} G(\rho ) & \mathrm{if}\;G\left(\rho \right)\leq G_{\max}\;\mathrm{and}\;\rho \leq R_{\mathrm{eff}}\\ G_{\max} & \mathrm{if}\;G\left(\rho \right)>G_{\max}\;\mathrm{and}\;\rho \leq R_{\mathrm{eff}}\\ 0 & \mathrm{otherwise} \end{array}\right.$$

Here, $G_{\max}$ is typically set to 1.5–2.0 (adjustable based on SNR), and $R_{\mathrm{eff}}$ denotes the effective imaging radius (slightly smaller than the array radius 9.to exclude the blind region of the probe).

#### 2.3.2. Analysis and Compensation Method of Inclination

**Figure 6:**
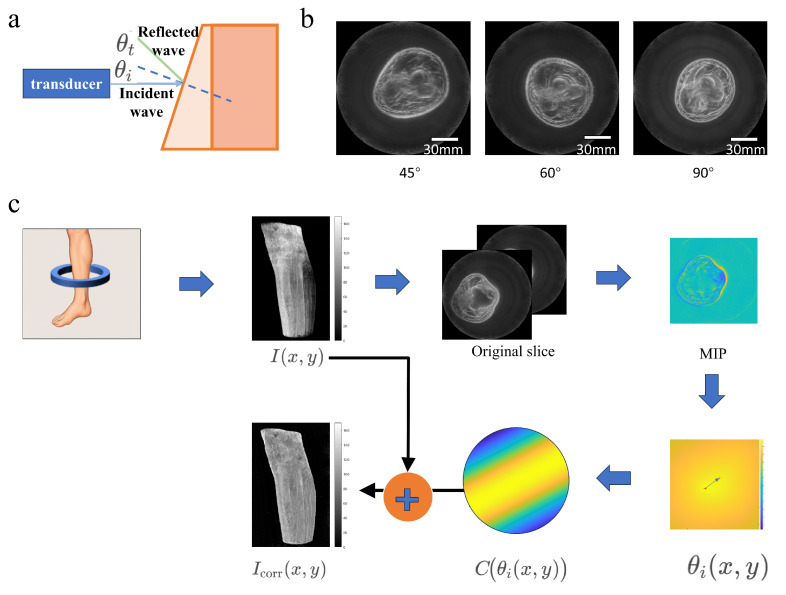
Analysis and Compensation Method of Inclination. Relationship of incident and reflected angles (**a**). USCT images of the forearm at 45°, 60° and 90° to the scanning plane (**b**). The detailed procedure of the Inclination Angle Compensation Method (**c**).

To correct for the USCT reflection image artifacts caused by “non-perpendicular incidence” (i.e., the inclination of the interface leads to the incident/transmitted energy deviating from the receiving direction and reducing the system’s detection efficiency, which in turn causes local echo intensity systematic bias), we adopt an incident angle-based gain correction algorithm. Figure 6 shows the analysis and compensation method of inclination effect. First, extract the surface contour of the imaging object from the multi-layer reflection images: For the slices below the target slice, perform maximum intensity projection (MIP), then obtain a stable foreground via threshold segmentation and morphological opening/closing operations. Next, use edge operators (e.g., Sobel) to extract the surface boundary and calculate its local slope along the tangential direction, thereby deriving the angle $\theta_{i}\left(x,y\right)$ between the surface normal and the direction of the acoustic wave. Perform two-dimensional interpolation, smoothing, and normalization of the angle field over the angular range, and generate an angular mask within the effective field of view (when the target boundary exits the FOV and the contour cannot be reliably estimated, the angular field is supplemented using adjacent lower slices). Subsequently, based on the longitudinal wave Snell’s law at the interface between the coupling medium and the tissue surface (e.g., skin), map the discrete $\theta_{i}$ to the transmission-related correction quantity: The refraction angle is given by Snell’s law:

(9) $$\frac{\sin\theta_{i}}{c_{i}}=\frac{\sin\theta_{t}}{c_{t}}\;\Rightarrow \;\theta_{t}=\arcsin\left(\frac{c_{t}}{c_{i}}\sin\theta_{i}\right),$$

where $c_{i}$ and $c_{t}$ are the sound velocities in the incident and transmission media, respectively. The pressure amplitude transmission coefficient at the interface is:

(10) $$T\left(\theta_{i}\right)=\frac{2z_{t}\cos\theta_{i}}{z_{t}\cos\theta_{i}+z_{i}\cos\theta_{t}},$$

where $z_{i}=\rho_{i}c_{i}$ and $z_{t}=\rho_{t}c_{t}$ are the acoustic impedances of the two media. To compensate for the transmission loss caused by oblique incidence, construct a gain function:

(11) $$G\left(\theta_{i}\right)=\frac{T(0)}{\max\left(T(\theta_{i}),\varepsilon \right)},$$

where ε is a stabilization term. If the image is processed in dB, the correction quantity is defined as:

(12) $$C\left(\theta_{i}\right)=20\log_{10}\left(G(\theta_{i})\right).$$

Finally, apply the correction to the original reflection image I(x,y):

(13) $$I_{\mathrm{corr}}\left(x,y\right)=I\left(x,y\right)+\kappa C\left(\theta_{i}\left(x,y\right)\right),$$

where κ is the correction intensity coefficient, used to control the compensation amplitude (to avoid saturation and noise elevation caused by over-compensation). κ can be automatically optimized on a set of representative slices by minimizing the “overflow rate” (the proportion of pixels exceeding the preset window width range after correction); meanwhile, an upper limit $G_{\max}$ is imposed on $G(\theta_{i})$, combined with an effective FOV mask, to suppress the amplification of edge noise and artifacts.

The key points of this correction framework are: using the surface contour to estimate the incident angle field, explicitly attributing the systematic intensity attenuation caused by oblique incidence to interface transmission loss, and performing pixel-level compensation on the reflection image with a physics-model-driven gain function, thereby improving the contrast and spatial uniformity of the reflection image; its main limitation arises when the surface echo is too weak or the incident angle is too large, where the contour extraction and angle estimation may fail, leading to under-correction.

### 2.4. Phantom Experiment

**Figure 7:**
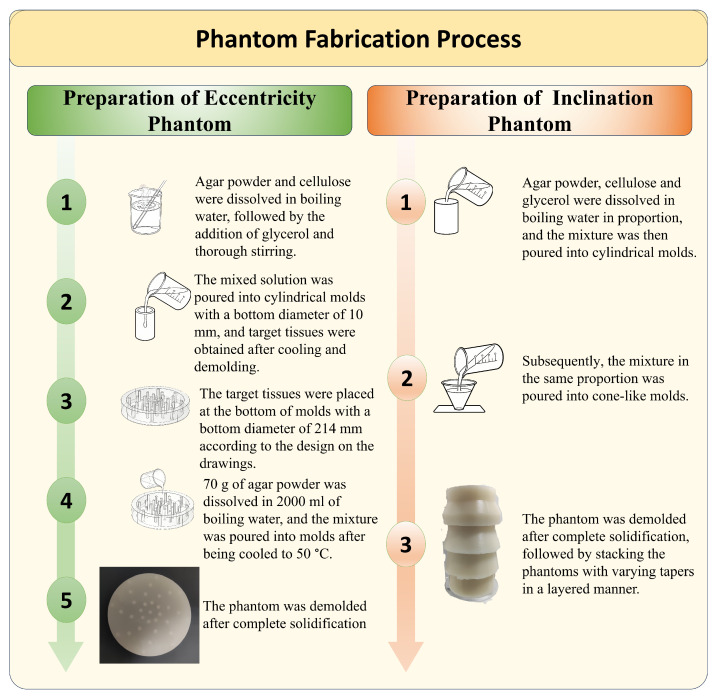
Fabrication Process of the Phantom. The left half of the figure shows the fabrication process of the eccentricity phantom, and the right half shows that of the inclination phantom.

A tissue-mimicking phantom was fabricated using agar powder (Sinopharm Chemical Reagent Co., Ltd., Shanghai, China), glycerol (Sinopharm Chemical Reagent Co., Ltd., Shanghai, China), and microcrystalline cellulose (Sinopharm Chemical Reagent Co., Ltd., Shanghai, China) to mimic the biomechanical and acoustic properties of human body-tissue structures.Experimental data were used to refine the simulation-based correction functions. To evaluate the effectiveness of the limb eccentricity correction algorithm, a cylindrical phantom with embedded targets was fabricated and imaged using a USCT device(Huazhong University of Science and Technology, Wuhan, China). This phantom was developed to compare the uniformity of echo intensity in images before and after correction. The phantom consists of an agar-based cylinder containing background tissue-mimicking material and embedded target inclusions. First, a solution containing cellulose scatterers (see Table 1) was poured into molds to form the target scattering structures. These were then positioned according to a reference point positioning diagram. Subsequently, the background tissue-mimicking solution was poured into the cylindrical mold to encapsulate the target scatterers [18]. After cooling and solidification, the phantom was placed at the center of the USCT scanning area for imaging. To quantitatively investigate the impact of limb-planes not being perfectly perpendicular to the scanning plane on echo intensity in imaging, a multi-angle phantom was designed and imaged using a USCT device. The tissue-mimicking phantom, composed of agar, glycerol, and cellulose, was shaped as multiple stacked cones to ensure uniform echo distribution across all directions under the annular probe. The oblique edges of the cones were angled at 0, 10, 20 and 30 degrees relative to the central axis, with the angle range studied spanning 0–30 degrees. This design accommodates the maximum curvature angle of limbs, which does not exceed 30 degrees. The phantom was placed on the scanning platform and positioned at the center of the scanning area. The probe scanned the phantom from top to bottom. After scanning, an appropriate sound velocity was set to reconstruct the image. Figure 7 shows the fabrication processes of those two phantoms.

## 3. Results

To comprehensively and quantitatively evaluate the improvement of imaging uniformity achieved by the proposed correction algorithm, four core quantitative evaluation metrics were introduced in the Section 3. These include the Radial Intensity Slope (RIS), the Center-to-Periphery Ratio (CPR), the Radial Non-Uniformity Index (RNU), and the Coefficient of Variation (CV). Specifically, the RIS is designed to quantify the linear attenuation trend of image intensity along the radial direction, where a larger RIS indicates a more pronounced intensity decay from the center to the periphery. The CPR serves as a direct metric for center-periphery contrast by comparing the average intensities of the central and peripheral regions, with a CPR value greater than 1 indicating a center-enhanced effect. The RNU characterizes the magnitude of radial intensity fluctuations across the entire field of view (FOV), with smaller RNU values corresponding to more uniform imaging. The CV measures the relative dispersion of intensity values; a higher CV indicates more significant overall intensity fluctuations, and it specifically reflects global variability without distinguishing between local and global features. By collectively employing these four metrics, the evolution of imaging uniformity can be systematically characterized from multiple dimensions, including the attenuation trend, the center-periphery contrast, the overall intensity fluctuation, and the relative degree of dispersion.

### 3.1. Validation of Eccentricity Effect

**Figure 8:**
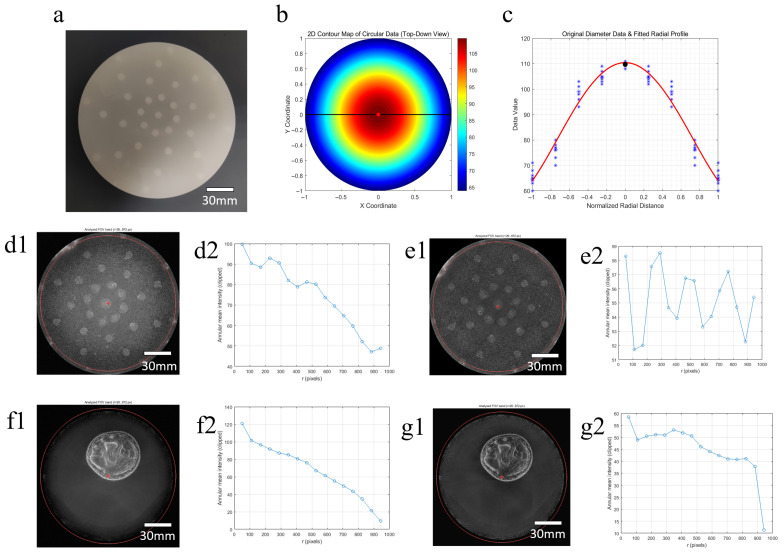
The clinical validation process of the eccentricity effect.Physical image of the eccentricity phantom (**a**); eccentricity error compensation function and the intensity attenuation trend from the edge to the center of the imaging region (**b**,**c**); direct imaging result of the phantom and the annular mean intensity versus radial distance curve (**d1**,**d2**); corrected imaging result of the phantom and the annular mean intensity versus radial distance curve, with consistent echo intensity of targets at different positions (**e1**,**e2**); imaging result of the human limb under the eccentric condition and the annular mean intensity versus radial distance curve, showing the phenomenon of “weaker at the edge and stronger at the center” (**f1**,**f2**); corrected imaging result of the human limb and the annular mean intensity versus radial distance curve, with favorable uniformity (**g1**,**g2**).

Figure 8 illustrates the validation process of the eccentricity effect. An eccentricity phantom embedded with uniformly scattering targets is first used for experiments. From the original imaging results of the phantom, an eccentricity error compensation function is extracted and quantified. The echo intensities of all targets are then statistically analyzed and fitted to derive the intensity attenuation trend from the edge to the center of the imaging region. After the original images are corrected based on this trend, the echo intensities of targets at different positions in the phantom images become consistent. Further human experiments verify the effectiveness of the method, as the corrected images eliminate eccentricity artifacts and achieve uniform echo intensity across the entire field of view.

The correction method was validated in both phantom and human experiments, with six different slices selected for parameter measurement in each group. In the phantom experiment (see Table 2), since the imaging object was a structurally uniform phantom, the measurement errors (standard deviations) of all metrics before and after algorithm correction were small, leading to highly consistent results. After correction, RIS, CPR, RNU, and CV all showed extremely significant decreases (*p* < 0.05), indicating that the eccentricity effect was effectively suppressed and imaging uniformity was greatly improved. In the human experiment (see Table 3), although the improvement of each metric was not as pronounced as in the phantom experiment, RIS, CPR, RNU, and CV also decreased significantly after correction (*p* < 0.05), with reductions of 72%, 53%, 34%, and 22%, respectively. This demonstrates that the proposed algorithm can still effectively enhance imaging uniformity in real clinical scenarios.

### 3.2. Validation of Inclination Effect

**Figure 9:**
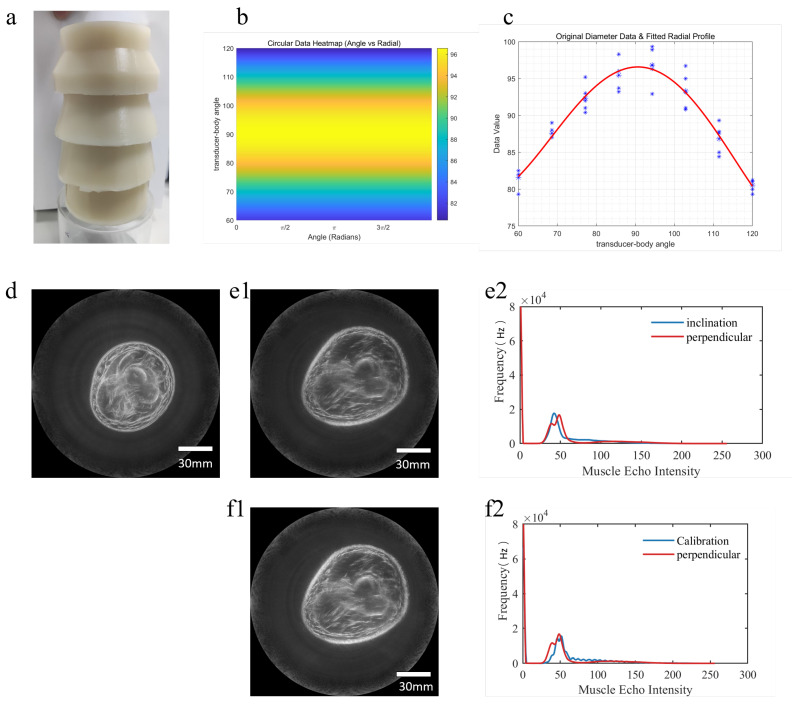
The validation process of the inclination angle effect.Physical image of the inclination phantom (**a**); inclination error compensation function and statistical analysis of echo intensities of the phantom at tilt angles from 60° to 120° (**b**,**c**); imaging result of the human limb in the vertical position, showing uniform echo intensity and high overall image brightness (**d**); imaging result of the same limb position at a 45° tilt (**e1**); pixel value histograms of imaging results in the vertical (red line) and 45° tilt (blue line) states, indicating an overall reduction in pixel values (**e2**); corrected image after inclination correction, with overall brightness restored compared to **e1** (**f1**); pixel histograms of the corrected image (blue line) and the vertical state (red line), demonstrating good consistency (**f2**).

Figure 9 shows the validation process of the inclination effect. An inclination phantom with circular targets of different tilt angles is first employed to simulate variations in echo intensity caused by different inclinations. From the original imaging results of the phantom, an inclination error compensation function for a specific angle is extracted. Echo intensities from the phantom at tilt angles ranging from 60° to 120° are then statistically analyzed and fitted with a polynomial to obtain the overall intensity trend. Human experiments further validate the method: when the limb is in a vertical position, the original image exhibits uniform echo intensity with high image quality; when the same limb is tilted at 45°, the original image shows an overall reduction in pixel values. After applying the correction method, the image quality of the tilted limb is restored, and the pixel value histogram of the corrected image aligns well with that of the vertical position, confirming the effectiveness of the inclination error correction in achieving consistent imaging performance.

It should be noted that the inclination correction can only improve a small part of the inclination-related errors, because the human limb cannot be tilted significantly in practical scenarios. Particularly in human experiments, the situation of large-angle limb inclination is extremely rare, and most are slight inclinations; as shown in the simulation part above, the impact of small-angle inclination on imaging effect is inherently small.

## 4. Discussion

Sarcopenia is a progressive muscular disorder characterized by loss of muscle mass and function, associated with increased adverse health outcomes including functional disability, falls, and mortality [19,20]. Tests such as CT and dual-energy X-ray absorptiometry (DXA), considered the gold standards for muscle mass assessment and sarcopenia diagnosis, are costly, have limited availability, require trained personnel, and expose patients to radiation. Therefore, there is a need to develop new methods to facilitate the diagnosis of sarcopenia.

This study proposes an echo intensity correction method for USCT. Increased echo intensity (EI) serves as an indicator of muscle degeneration, manifested as an elevated proportion of fat and connective tissue within muscles [21]. Numerous studies have confirmed that lower limb muscle echo intensity aids in the diagnosis of sarcopenia [22,23,24]. However, its assessment may be influenced by various external factors, such as ultrasound probe parameters, probe inclination, patient rest time, subject positioning, and patient hydration status [25]. Scafoglieri et al. demonstrated that even after gain, depth, and frequency normalization, EI values still exhibited significant differences across additional ultrasound settings [26].

To minimize the influence of external factors, we systematically identified and compensated for key error factors such as inclination effect and eccentricity effect, significantly improving the accuracy and reliability of echo intensity measurement in sarcopenia diagnosis.

For the first time, we established the quantitative relationship between probe inclination angle, limb eccentricity and echo intensity through a combined approach of finite element simulation and biomimetic model experiments. The calibration functions of these two factors exhibited significant nonlinear relationships with echo intensity. Notably, eccentricity accounted for up to 70% of the intensity uniformity within the same slice, while the inclination angle primarily contributed to intensity variations between different slices (approximately 30%).

This finding is highly consistent with the physical mechanism of USCT: The central superposition effect of the acoustic field in the annular probe is exacerbated by limb eccentricity, leading to uneven intensity distribution, while changes in inclination angle result in signal attenuation by altering the incidence direction of acoustic waves. To correct these effects, we extracted actual eccentricity and inclination parameters, followed by two-stage compensation of initial echo intensity through querying calibration functions: eccentricity correction prioritizes restoring intensity uniformity within single-layer images, while inclination correction optimizes interlayer consistency. In the corrected images, the intensity gradients originally caused by eccentricity or inclination are significantly reduced, and the echo distribution at the skin–muscle interface becomes more uniform, validating the effectiveness of this method.

Validation on imaging data from volunteer was also conducted in the study. We performed imaging on one healthy subject and one volunteer with sarcopenia. After echo intensity correction, clear visualization of muscle, connective tissue, and skeletal features was achieved, demonstrating high compatibility in the detection of muscle atrophy.

Compared to conventional methods that rely solely on mean echo intensity or single-factor correction, the multivariate correction framework in this study more comprehensively reflects the complex acoustic properties of muscle tissue.

Limitations and Future: Although this study has validated the reliability of the correction curve through biomimetic model simulations, its diagnostic efficacy has not yet been extensively tested in clinical populations. For the diagnosis of musculoskeletal (MSK) diseases in future research, we will recruit more clinically relevant subjects, including healthy control groups and volunteers with MSK diseases, and strive to further improve the diagnostic accuracy of the proposed method through in-depth and systematic clinical validation. Meanwhile, future research should focus on optimizing the calibration model for different pathological conditions (e.g., muscle atrophy, adipose infiltration). Additionally, the current algorithm may have limitations in analyzing images of deep muscle or obese patients, and subsequent work could incorporate deep learning techniques to enhance the robustness of parameter extraction.

## 5. Conclusions

We developed an echo intensity correction method for USCT that compensates for probe tilt, limb eccentricity, and tissue anisotropy. The method significantly enhances the accuracy and reproducibility of echo intensity measurements, making USCT a more reliable tool for quantitative MSK disease diagnosis.

## Figures and Tables

**Table 1:** Formulation components and proportion of eccentricity phantom preparation (per 100 mL water).

Ingredient	Target Tissues Material	Background Tissues Material
agar power	3.5 g	3.5 g
cellulose	1.5 g	-
glycerol	8 mL	-

**Table 2:** Results of phantom experiments.

Metric	Before (Mean ± SD)	After (Mean ± SD)	*p*-Value
Radial Intensity Slope (RIS)	0.05612 ± 0.00023	0.000818 ± 0.000012	<0.05
Center-to-Periphery Ratio (CPR)	1.660 ± 0.018	0.985 ± 0.004	<0.05
Radial Non-Uniformity (RNU)	0.702 ± 0.005	0.127 ± 0.003	<0.05
Coefficient of Variation (CV)	0.267 ± 0.002	0.173 ± 0.002	<0.05

**Table 3:** Results of human experiments.

Metric	Before (Mean ± SD)	After (Mean ± SD)	Reduction	*p*-Value
RIS	0.106 ± 0.010	0.0296 ± 0.0001	72% ↓	<0.05
CPR	2.776 ± 0.063	1.313 ± 0.017	53% ↓	<0.05
RNU	1.583 ± 0.057	1.044 ± 0.008	34% ↓	<0.05
CV	0.629 ± 0.011	0.491 ± 0.007	22% ↓	<0.05

↓: indicates a reduction in the corresponding metric.

## Data Availability

The original contributions presented in this study are included in the article. Further inquiries can be directed to the corresponding authors.

## References

[1] Vos T., Lim S.S., Abbafati C., Abbas K.M., Abbasi M., Abbasifard M., Abbasi-Kangevari M., Abbastabar H., Abd-Allah F., Abdelalim A. (2020). Global Burden of 369 Diseases and Injuries in 204 Countries and Territories, 1990–2019: A Systematic Analysis for the Global Burden of Disease Study 2019. Lancet.

[2] World Health Organization (WHO) Musculoskeletal Health. https://www.who.int/news-room/fact-sheets/detail/musculoskeletal-conditions.

[3] Chen L.K., Liu L.K., Woo J., Assantachai P., Auyeung T.W., Bahyah K.S., Chou M., Chen L., Hsu P., Krairit O. (2014). Sarcopenia in Asia: Consensus Report of the Asian Working Group for Sarcopenia. J. Am. Med. Dir. Assoc..

[4] Xu W., Chen T., Cai Y., Hu Y., Fan L., Wu C. (2020). Sarcopenia in Community-Dwelling Oldest Old Is Associated with Disability and Poor Physical Function. J. Nutr. Health Aging.

[5] Petermann-Rocha F., Balntzi V., Gray S.R., Lara J., Ho F.K., Pell J.P., Celis-Morales C. (2022). Global Prevalence of Sarcopenia and Severe Sarcopenia: A Systematic Review and Meta-Analysis. J. Cachexia Sarcopenia Muscle.

[6] Shin Y., Yang J., Lee Y.H., Kim S. (2021). Artificial Intelligence in Musculoskeletal Ultrasound Imaging. Ultrasonography.

[7] Shinohara I., Inui A., Mifune Y., Nishimoto H., Yamaura K., Mukohara S., Yoshikawa T., Kato T., Furukawa T., Hoshino Y. (2022). Motion Analysis of Triangular Fibrocartilage Complex by Using Ultrasonography Images: Preliminary Analysis. Sensors.

[8] Özçakar L., Ata A.M., Kaymak B., Kara M., Kumbhare D. (2018). Ultrasound Imaging for Sarcopenia, Spasticity and Painful Muscle Syndromes. Curr. Opin. Support. Palliat. Care.

[9] Wu J., Luo H., Ren S., Shen L., Cheng D., Wang N. (2022). Enhanced Echo Intensity of Skeletal Muscle Is Associated with Poor Physical Function in Hemodialysis Patients: A Cross-Sectional Study. BMC Nephrol..

[10] Mikołajowski G., Pałac M., Wolny T., Linek P. (2021). Lateral Abdominal Muscles Shear Modulus and Thickness Measurements under Controlled Ultrasound Probe Compression by External Force Sensor: A Comparison and Reliability Study. Sensors.

[11] Iqbal I., Younus M., Walayat K., Kakar M.U., Ma J. (2021). Automated multi-class classification of skin lesions through deep convolutional neural network with dermoscopic images. Comput. Med. Imaging Graph..

[12] Iqbal I. (2020). Deep learning-based automated detection of human knee joint’s synovial fluid from magnetic resonance images with transfer learning. IET Image Process..

[13] Ali R., Mitcham T.M., Brevett T., Agudo O.C., Martinez C.D., Li C., Doyley M.M., Duric N. (2024). 2-D Slicewise Waveform Inversion of Sound Speed and Acoustic Attenuation for Ring Array Ultrasound Tomography Based on a Block LU Solver. IEEE Trans. Med. Imaging.

[14] Kim Y.J., Choi J., Moon J., Sung K.R., Choi J. (2021). A Sarcopenia Detection System Using an RGB-D Camera and an Ultrasound Probe: Eye-in-Hand Approach. Biosensors.

[15] Song J., Zhang Q., Zhou L., Quan Z., Wang S., Liu Z. (2021). Design and implementation of a modular and scalable research platform for ultrasound computed tomography. IEEE Trans. Ultrason. Ferroelectr. Freq. Control.

[16] Liu Z., Zhou X., Yang H., Zhang Q., Zhou L., Wu Y., Liu Q., Yan W., Song J., Ding M. (2025). Reconstruction of reflection ultrasound computed tomography with sparse transmissions using conditional generative adversarial network. Ultrasonics.

[17] Savitzky A., Golay M.J.E. (1964). Smoothing and differentiation of data by simplified least squares procedures. Anal. Chem..

[18] du Toit C., Dima R., Papernick S., Jonnalagadda M., Tessier D., Fenster A., Lalone E. (2024). Three-dimensional ultrasound to investigate synovitis in first carpometacarpal osteoarthritis: A feasibility study. Med. Phys..

[19] Kim T.N., Park M.S., Yang S.J., Yoo H.J., Kang H.J., Song W., Seo J.A., Kim S.G., Kim N.H., Baik S.H. (2010). Prevalence and Determinant Factors of Sarcopenia in Patients with Type 2 Diabetes: The Korean Sarcopenic Obesity Study (KSOS). Diabetes Care.

[20] Wolfson L., Judge J., Whipple R., King M. (1995). Strength Is a Major Factor in Balance, Gait, and the Occurrence of Falls. J. Gerontol. A Biol. Sci. Med. Sci..

[21] Wilhelm E.N., Rech A., Minozzo F., Radaelli R., Botton C.E., Pinto R.S. (2014). Relationship between Quadriceps Femoris Echo Intensity, Muscle Power, and Functional Capacity of Older Men. Age.

[22] Watanabe Y., Ikenaga M., Yoshimura E., Yamada Y., Kimura M. (2018). Association between Echo Intensity and Attenuation of Skeletal Muscle in Young and Older Adults: A Comparison between Ultrasonography and Computed Tomography. Clin. Interv. Aging.

[23] Isaka M., Sugimoto K., Yasunobe Y., Akasaka H., Fujimoto T., Kurinami H., Takeya Y., Yamamoto K., Rakugi H. (2019). The Usefulness of an Alternative Diagnostic Method for Sarcopenia Using Thickness and Echo Intensity of Lower Leg Muscles in Older Males. J. Am. Med. Dir. Assoc..

[24] Yamada M., Kimura Y., Ishiyama D., Nishio N., Abe Y., Kakehi T., Fujimoto J., Tanaka T., Ohji S., Otobe Y. (2017). Differential Characteristics of Skeletal Muscle in Community-Dwelling Older Adults. J. Am. Med. Dir. Assoc..

[25] Formenti P., Umbrello M., Coppola S., Froio S., Chiumello D. (2019). Clinical Review: Peripheral Muscular Ultrasound in the ICU. Ann. Intensive Care.

[26] Scafoglieri A., Van den Broeck J., Bartocci P., Cattrysse E., Jager-Wittenaar H., Gonzalez M.C. (2024). Skeletal Muscle Echo Intensity Values Differ Significantly across Ultrasound Parameter Settings. Life.

